# Supplementary material for: Pyruvate Dehydrogenase Kinase 1 inhibition mediated oxidative phosphorylation enhancement in cartilage promotes osteoarthritis progression
Source: BMC Musculoskelet Disord. 2023 Jul 20;24:597. doi: 10.1186/s12891-023-06585-6 (PMC10357736; doi:10.1186/s12891-023-06585-6)
Supplement: Supplementary file 1 — Additional file 1. [file 12891_2023_6585_MOESM1_ESM.zip › MF.Gsea.1653627316242/GOMF_DNA_HELICASE_ACTIVITY.html]

Details for gene set GOMF\_DNA\_HELICASE\_ACTIVITY[GSEA]

|  || Dataset | 55457\_collapsed\_to\_symbols.cls.cls #OA\_versus\_normal.cls.cls #OA\_versus\_normal\_repos |
| Phenotype | cls.cls#OA\_versus\_normal\_repos |
| Upregulated in class | normal |
| GeneSet | GOMF\_DNA\_HELICASE\_ACTIVITY |
| Enrichment Score (ES) | -0.39489764 |
| Normalized Enrichment Score (NES) | -1.5535153 |
| Nominal p-value | 0.017828202 |
| FDR q-value | 0.15601613 |
| FWER p-Value | 1.0 |
Table: GSEA Results Summary

  

Fig 1: Enrichment plot: GOMF\_DNA\_HELICASE\_ACTIVITY      
 Profile of the Running ES Score & Positions of GeneSet Members on the Rank Ordered List

  

| SYMBOL | TITLE | RANK IN GENE LIST | RANK METRIC SCORE | RUNNING ES | CORE ENRICHMENT || 1 | MCM5 | minichromosome maintenance complex component 5 [Source:HGNC Symbol;Acc:HGNC:6948] | 351 | 0.724 | 0.0059 | No |
| 2 | RFC3 | replication factor C subunit 3 [Source:HGNC Symbol;Acc:HGNC:9971] | 570 | 0.633 | 0.0179 | No |
| 3 | MCM2 | minichromosome maintenance complex component 2 [Source:HGNC Symbol;Acc:HGNC:6944] | 888 | 0.546 | 0.0183 | No |
| 4 | RFC4 | replication factor C subunit 4 [Source:HGNC Symbol;Acc:HGNC:9972] | 897 | 0.543 | 0.0426 | No |
| 5 | XRCC5 | X-ray repair cross complementing 5 [Source:HGNC Symbol;Acc:HGNC:12833] | 1018 | 0.513 | 0.0568 | No |
| 6 | MCM6 | minichromosome maintenance complex component 6 [Source:HGNC Symbol;Acc:HGNC:6949] | 1131 | 0.490 | 0.0705 | No |
| 7 | RFC2 | replication factor C subunit 2 [Source:HGNC Symbol;Acc:HGNC:9970] | 1247 | 0.467 | 0.0830 | No |
| 8 | CHD1L | chromodomain helicase DNA binding protein 1 like [Source:HGNC Symbol;Acc:HGNC:1916] | 1320 | 0.454 | 0.0983 | No |
| 9 | RAD51 | RAD51 recombinase [Source:HGNC Symbol;Acc:HGNC:9817] | 1581 | 0.416 | 0.0971 | No |
| 10 | DSCC1 | DNA replication and sister chromatid cohesion 1 [Source:HGNC Symbol;Acc:HGNC:24453] | 2362 | 0.319 | 0.0510 | No |
| 11 | RUVBL2 | RuvB like AAA ATPase 2 [Source:HGNC Symbol;Acc:HGNC:10475] | 2778 | 0.272 | 0.0312 | No |
| 12 | ERCC2 | "ERCC excision repair 2, TFIIH core complex helicase subunit [Source:HGNC Symbol;Acc:HGNC:3434]" | 3544 | 0.198 | -0.0193 | No |
| 13 | MCM3 | minichromosome maintenance complex component 3 [Source:HGNC Symbol;Acc:HGNC:6945] | 3675 | 0.188 | -0.0208 | No |
| 14 | POLQ | DNA polymerase theta [Source:HGNC Symbol;Acc:HGNC:9186] | 4293 | 0.129 | -0.0629 | No |
| 15 | UPF1 | UPF1 RNA helicase and ATPase [Source:HGNC Symbol;Acc:HGNC:9962] | 4434 | 0.118 | -0.0684 | No |
| 16 | CHD9 | chromodomain helicase DNA binding protein 9 [Source:HGNC Symbol;Acc:HGNC:25701] | 4520 | 0.112 | -0.0699 | No |
| 17 | ASCC3 | activating signal cointegrator 1 complex subunit 3 [Source:HGNC Symbol;Acc:HGNC:18697] | 4689 | 0.097 | -0.0785 | No |
| 18 | XRCC6 | X-ray repair cross complementing 6 [Source:HGNC Symbol;Acc:HGNC:4055] | 4699 | 0.096 | -0.0748 | No |
| 19 | BLM | BLM RecQ like helicase [Source:HGNC Symbol;Acc:HGNC:1058] | 4843 | 0.084 | -0.0821 | No |
| 20 | RAD54B | RAD54 homolog B [Source:HGNC Symbol;Acc:HGNC:17228] | 5753 | 0.012 | -0.1524 | No |
| 21 | BRIP1 | BRCA1 interacting helicase 1 [Source:HGNC Symbol;Acc:HGNC:20473] | 5885 | 0.003 | -0.1624 | No |
| 22 | MCM4 | minichromosome maintenance complex component 4 [Source:HGNC Symbol;Acc:HGNC:6947] | 6003 | -0.005 | -0.1713 | No |
| 23 | RAD50 | RAD50 double strand break repair protein [Source:HGNC Symbol;Acc:HGNC:9816] | 6474 | -0.040 | -0.2061 | No |
| 24 | RUVBL1 | RuvB like AAA ATPase 1 [Source:HGNC Symbol;Acc:HGNC:10474] | 7477 | -0.116 | -0.2788 | No |
| 25 | ATRX | ATRX chromatin remodeler [Source:HGNC Symbol;Acc:HGNC:886] | 7551 | -0.123 | -0.2788 | No |
| 26 | RAD54L2 | RAD54 like 2 [Source:HGNC Symbol;Acc:HGNC:29123] | 7872 | -0.146 | -0.2970 | No |
| 27 | ERCC6 | "ERCC excision repair 6, chromatin remodeling factor [Source:HGNC Symbol;Acc:HGNC:3438]" | 7939 | -0.150 | -0.2953 | No |
| 28 | SETX | senataxin [Source:HGNC Symbol;Acc:HGNC:445] | 8398 | -0.185 | -0.3224 | No |
| 29 | ERCC3 | "ERCC excision repair 3, TFIIH core complex helicase subunit [Source:HGNC Symbol;Acc:HGNC:3435]" | 8669 | -0.206 | -0.3340 | No |
| 30 | MCM9 | minichromosome maintenance 9 homologous recombination repair factor [Source:HGNC Symbol;Acc:HGNC:21484] | 8781 | -0.215 | -0.3328 | No |
| 31 | CHD4 | chromodomain helicase DNA binding protein 4 [Source:HGNC Symbol;Acc:HGNC:1919] | 8917 | -0.226 | -0.3330 | No |
| 32 | RFC5 | replication factor C subunit 5 [Source:HGNC Symbol;Acc:HGNC:9973] | 9499 | -0.274 | -0.3656 | No |
| 33 | GTF2F2 | general transcription factor IIF subunit 2 [Source:HGNC Symbol;Acc:HGNC:4653] | 9876 | -0.309 | -0.3807 | Yes |
| 34 | WRN | WRN RecQ like helicase [Source:HGNC Symbol;Acc:HGNC:12791] | 10010 | -0.323 | -0.3763 | Yes |
| 35 | MCM7 | minichromosome maintenance complex component 7 [Source:HGNC Symbol;Acc:HGNC:6950] | 10032 | -0.325 | -0.3630 | Yes |
| 36 | RECQL | RecQ like helicase [Source:HGNC Symbol;Acc:HGNC:9948] | 10124 | -0.333 | -0.3548 | Yes |
| 37 | RECQL5 | RecQ like helicase 5 [Source:HGNC Symbol;Acc:HGNC:9950] | 10144 | -0.335 | -0.3409 | Yes |
| 38 | IGHMBP2 | immunoglobulin mu DNA binding protein 2 [Source:HGNC Symbol;Acc:HGNC:5542] | 10248 | -0.346 | -0.3331 | Yes |
| 39 | CHD3 | chromodomain helicase DNA binding protein 3 [Source:HGNC Symbol;Acc:HGNC:1918] | 10269 | -0.347 | -0.3187 | Yes |
| 40 | CHD2 | chromodomain helicase DNA binding protein 2 [Source:HGNC Symbol;Acc:HGNC:1917] | 10467 | -0.370 | -0.3171 | Yes |
| 41 | DHX9 | DExH-box helicase 9 [Source:HGNC Symbol;Acc:HGNC:2750] | 10627 | -0.386 | -0.3118 | Yes |
| 42 | WRNIP1 | WRN helicase interacting protein 1 [Source:HGNC Symbol;Acc:HGNC:20876] | 10909 | -0.418 | -0.3145 | Yes |
| 43 | G3BP1 | G3BP stress granule assembly factor 1 [Source:HGNC Symbol;Acc:HGNC:30292] | 11117 | -0.445 | -0.3101 | Yes |
| 44 | MRE11 | "MRE11 homolog, double strand break repair nuclease [Source:HGNC Symbol;Acc:HGNC:7230]" | 11458 | -0.497 | -0.3138 | Yes |
| 45 | NAV2 | neuron navigator 2 [Source:HGNC Symbol;Acc:HGNC:15997] | 11619 | -0.520 | -0.3025 | Yes |
| 46 | ERCC6L | "ERCC excision repair 6 like, spindle assembly checkpoint helicase [Source:HGNC Symbol;Acc:HGNC:20794]" | 11663 | -0.527 | -0.2817 | Yes |
| 47 | CHD5 | chromodomain helicase DNA binding protein 5 [Source:HGNC Symbol;Acc:HGNC:16816] | 11776 | -0.545 | -0.2654 | Yes |
| 48 | CHD8 | chromodomain helicase DNA binding protein 8 [Source:HGNC Symbol;Acc:HGNC:20153] | 11825 | -0.554 | -0.2437 | Yes |
| 49 | RECQL4 | RecQ like helicase 4 [Source:HGNC Symbol;Acc:HGNC:9949] | 11927 | -0.578 | -0.2251 | Yes |
| 50 | RTEL1 | regulator of telomere elongation helicase 1 [Source:HGNC Symbol;Acc:HGNC:15888] | 11968 | -0.587 | -0.2012 | Yes |
| 51 | TWNK | twinkle mtDNA helicase [Source:HGNC Symbol;Acc:HGNC:1160] | 12069 | -0.612 | -0.1810 | Yes |
| 52 | SUPV3L1 | Suv3 like RNA helicase [Source:HGNC Symbol;Acc:HGNC:11471] | 12087 | -0.616 | -0.1540 | Yes |
| 53 | DNA2 | DNA replication helicase/nuclease 2 [Source:HGNC Symbol;Acc:HGNC:2939] | 12148 | -0.631 | -0.1298 | Yes |
| 54 | RAD54L | RAD54 like [Source:HGNC Symbol;Acc:HGNC:9826] | 12204 | -0.646 | -0.1044 | Yes |
| 55 | CHD7 | chromodomain helicase DNA binding protein 7 [Source:HGNC Symbol;Acc:HGNC:20626] | 12591 | -0.791 | -0.0982 | Yes |
| 56 | DDX11 | DEAD/H-box helicase 11 [Source:HGNC Symbol;Acc:HGNC:2736] | 12607 | -0.803 | -0.0625 | Yes |
| 57 | CHD1 | chromodomain helicase DNA binding protein 1 [Source:HGNC Symbol;Acc:HGNC:1915] | 12745 | -0.908 | -0.0316 | Yes |
| 58 | DDX3X | DEAD-box helicase 3 X-linked [Source:HGNC Symbol;Acc:HGNC:2745] | 12788 | -0.949 | 0.0087 | Yes |
Table: GSEA details [plain text format]

  

Fig 2: GOMF\_DNA\_HELICASE\_ACTIVITY      
 Blue-Pink O' Gram in the Space of the Analyzed GeneSet

  

Fig 3: GOMF\_DNA\_HELICASE\_ACTIVITY: Random ES distribution      
 Gene set null distribution of ES for **GOMF\_DNA\_HELICASE\_ACTIVITY**
